# Gut microbiome dynamics and cardiometabolic disease progression: evidence from a secondary data analysis of pooled clinical datasets

**DOI:** 10.3389/fmicb.2026.1790805

**Published:** 2026-07-27

**Authors:** Scott D. Wrigley, Sarah A. Johnson, Christopher L. Gentile, Tiffany L. Weir

**Affiliations:** 1 Intestinal Health Lab, Department of Food Science and Human Nutrition, Colorado State University, Fort Collins, CO, United States; 2 Integrative Cardiovascular Physiology Lab, Department of Food Science and Human Nutrition, Colorado State University, Fort Collins, CO, United States; 3 Department of Health, Nutrition, and Food Sciences, Florida State University, Tallahassee, FL, United States

**Keywords:** cardiovascular disease, dyslipidemia, endothelial function, gut microbiome, hypertension, microbial networks

## Abstract

**Purpose:**

Individuals with cardiometabolic disease (CMD) often exhibit decreased microbial alpha diversity and/or differences in beta diversity indices than those without CMD. However, it is unclear if these compositional changes in the gut microbiome are a cause or a consequence of CMD. Research suggests individual bacterial species act as drivers of disease, inducing shifts in microbial community and host metabolism. During this process, large-scale compositional changes can develop secondarily, obscuring the original microbial drivers. This study aimed to characterize the gut microbiota of healthy individuals compared to those with early risk factors for CMD to determine whether specific microbial taxa and community associations exist with early stages of hypertension, vascular dysfunction, dyslipidemia, and overweight/obesity.

**Procedure:**

Baseline anthropometric, physiological, and gut microbiome data from three clinical studies previously conducted by our research groups were compiled and re-analyzed.

**Results:**

No differences in alpha and/or beta diversity were observed across CMD parameters. Through a consensus-based differential abundance analysis, we observed that several health-related taxa decreased as CMD levels increased, including *Akkermansia, Bacteroides, Bifidobacterium, Blautia, Eubacterium, Lachnospiraceae, Oscillospiraceae, Prevotella, Roseburia,* and *Ruminococcus.* Furthermore, co-occurrence networks of individuals with elevated cardiometabolic parameters showed lower clustering coefficients, higher path lengths, lower degrees, higher modularity, and higher negative cohesion than those with normal parameters.

**Implications:**

The loss of health-associated gut microbiota, along with decreased network connectivity and increased network fragmentation, may play a role in the progression of CMD.

## 1 Introduction

Cardiovascular disease (CVD) continues to grow unabated worldwide. Between 1990 and 2020, CVD deaths increased from 12.1 million to 19 million annually, a ~57% increase over 30 years (Roth et al., 2020; Tsao et al., 2022; WHO, 2017). Despite significant advances in science and medicine, CVD-related mortalities are projected to rise to 24 million per year by 2030 (Jones and Greene, 2012; Bansilal et al., 2015; Angell et al., 2020), a ~26% increase in a single decade. In part, the continued growth of the global CVD burden is due to the increasing prevalence of cardiometabolic disease (CMD), including disorders such as hypertension, vascular dysfunction, dyslipidemia, and obesity (Du and Qin, 2023; Fuchs and Whelton, 2020; Sun H. J. et al., 2019; Powell-Wiley et al., 2021).

Although the causes of these risk factors are complex, research supports the role of gut microbiota in their development. Germ-free mice, fecal microbiota transplantation, and antibiotic studies indicate that gut microbiota contribute to the development of hypertension, dyslipidemia, vascular dysfunction, and obesity. For example, our group (Battson et al., 2018a), along with others (Vikram et al., 2016), have demonstrated that suppressing the gut microbiota with broad-spectrum antibiotics can restore diet-induced vascular dysfunction in experimental mice. We have also reported that transplanting gut microbiota from obese mice or humans was sufficient to induce vascular dysfunction in lean mice (Battson et al., 2019; Trikha et al., 2021). In a series of elegant experiments, Wu and colleagues showed that individuals with obesity had a higher proportion of *Megamonas* spp., and that polygenic risk scores and *Megamonas* contributed additively to obesity (Wu et al., 2024). Furthermore, transplantation of *Megamonas rupellensis* into germ-free mice promoted obesity by enhancing lipid absorption via myo-inositol degradation. Correspondingly, transplanting the gut microbiota from individuals with hypertension (Li et al., 2017) or dyslipidemia (Xu et al., 2023) into germ-free mice increased blood pressure and blood lipid levels, respectively. Taken together, these observations suggest that the gut microbiota plays a causal role in the development of hypertension, dyslipidemia, vascular dysfunction, and obesity.

Despite this evidence, the precise mechanisms by which alterations in the gut microbiota facilitate the development of CMD remain elusive. Compared with healthy controls, the gut microbiota of individuals with cardiometabolic risk factors is frequently characterized by decreased alpha diversity and/or differences in beta diversity (Lopez-Montoya et al., 2022; Pinart et al., 2021; Sun S. et al., 2019; Louca et al., 2021; Fei et al., 2019). However, alpha-diversity measures are often equivocal, with some research reporting differences between healthy individuals and those with CMD, while others report no difference. Furthermore, despite differences in beta-diversity, no consistent taxa changes associated with these parameters have been reported.

Recent research suggests that individual taxa with unique functional capacities, rather than large-scale shifts in diversity, may be drivers of diseases processes. However, these drivers may be obscured by community-level shifts that occur secondary to, and as a result of, driver bacteria. In 2011, based on their work examining the role of enterotoxigenic *Bacteroides fragilis* (ETBF) in colorectal cancer, Sears and Pardoll posited the Alpha-bug hypothesis (Sears and Pardoll, 2011), wherein unique traits enable specific driver bacteria to initiate disease and remodel the gut microbiota, further promoting disease progression. Subsequently, in 2012, Tjalsma and colleagues expanded on the Alpha-bug hypothesis with the Driver-Passenger hypothesis, a concept based in ecological theory (Tjalsma et al., 2012). As in the Alpha-bug hypothesis, driver bacteria (alpha-bugs) may initiate disease and induce shifts in the gut microbiota community. However, subsequent changes in host physiology caused by the nascent disease state also alter the gut microbial ecosystem. Bacteria that are more suited to thrive in the nascent disease microbiome (passengers) may outcompete other bacteria for resources, including the drivers. Therefore, by the time large-scale diversity-altering compositional changes are identified in overt disease, it may be too late to identify driver bacteria and the subtler microbial community changes, such as changes in network connectivity, that initiated disease progression.

By applying this concept to CMD, we attempted to identify driver bacteria that may be obscured in overt disease by examining gut microbiota profiles in individuals ranging from metabolically healthy through early stages of CMD progression. Furthermore, by stratifying these risk factors, we could explore whether there were patterns in microbial community interactions associated with increasing risk progression. To achieve this, we compiled baseline 16S rRNA data from participants of three previous clinical studies conducted by our laboratories. These individuals were generally free of overt disease, but subsets within each dataset were beginning to develop forms of CMD, including elevated blood pressure, vascular dysfunction, dyslipidemia, and elevated body mass index (BMI). We hypothesized that large-scale compositional differences, characterized by alpha and beta-diversity indices, would not be apparent in individuals with pre-clinical stages of metabolic disturbance compared to healthy individuals. However, we predicted that there would be driver bacteria and shifts in co-occurrence networks that may contribute to altered microbial community structure often apparent in later stages of CMD. Contrary to our hypothesis, we did not identify specific drivers of CMD progression. Instead, we observed a loss of health-associated taxa, such as bacteria that produce short-chain fatty acids (SCFAs), as well as remodeling of co-occurrence networks, which may indicate transitory states between health and CMD-associated microbial communities.

## 2 Materials and methods

### 2.1 Study design

This study represents a secondary analysis of baseline data from three previous clinical trials conducted by our research groups. Baseline anthropometric, physiological, and gut microbiome data were compiled and analyzed to identify potential differences in microbial communities between subjects manifesting CMD, including gradations of dyslipidemia, hypertension, vascular dysfunction, and body mass index (BMI).

### 2.2 Participant characteristics

Participants were recruited from the Fort Collins, Colorado area via email, social media, flyers, direct mailers, and/or word of mouth. Participants underwent a phone interview for eligibility prescreening, and those who met the inclusion criteria were scheduled for an on-site screening visit to confirm their inclusion or exclusion status. Of note, although there were differences in age and sex criteria for inclusion or exclusion, all three studies excluded subjects with health conditions or on medications that could influence the study outcomes (i.e., cardiometabolic endpoints). Participant recruitment and inclusion criteria for the Phage 2 (NCT04511221) and Microgreens (NCT04239898) studies were as previously described (Grubb et al., 2020; Lee et al., 2025). The recruitment and exclusion criteria for the Aronia Berry study (NCT03824041) were as follows: men and postmenopausal women (> 1 year from cessation of menstruation), 45–75 years of age, with baseline endothelial dysfunction (RHI ≤ 1.67), hemoglobin A1C ≤ 6.4%, blood pressure < 129/80 mmHg, total cholesterol < 240 mg/dL, LDL cholesterol < 190 mg/dL, triglycerides < 350 mg/dL, body mass index ≥ 18.5 and < 30 kg/m2, and willing to maintain their normal eating/drinking habits and exercise habits to avoid changes in body weight over the duration of the study were invited to participate. Exclusion criteria included: taking antihypertensive, lipid-lowering, and/or hormone replacement medications; diagnosed hypertension, CVD, diabetes, metabolic syndrome, cancer, kidney, liver, and/or pancreatic disease; obese participants, defined as BMI superior or equal to 30; neuropathy, thrombosis, or past arm trauma or surgery; > 3 days/wk. vigorous exercise; participating in a weight loss program; weight change > 5% in the past 3 months; current smokers or history of smoking in the last 12 months; current smokers or history of smoking in the last 12 months; antibiotic use at any point during the study or 2 months prior to enrollment; allergies to aronia berries or other study materials; and unwillingness to maintain normal diet and/or physical activity pattern, or to discontinue use of dietary supplements for the duration of the study. A summary of baseline participant characteristics can be found in Table 1.

**Table 1:** Mean participant characteristics in each study, and as one cohort (total).

Study	Female	Male	Age	Systolic pressure (mmHg)	Diastolic pressure (mmHg)	Reactive hyperemia index	Low density lipoprotein	Total cholesterol	Body mass index
Aronia Berry	10	12	57.3 ± 6.2	112.3 ± 10.1	71.0 ± 8.7	2.1 ± 0.6	113.4 ± 25.6	193.8 ± 30.1	24.6 ± 2.9
Microgreens	15	8	57.2 ± 6.2	118.7 ± 10.3	71.5 ± 7.8	-	111.5 ± 16.2	202.0 ± 18.5	23.9 ± 2.4
Phage 2	55	32	37.0 ± 13.0	118.0 ± 11.3	70.8 ± 8.6	2.2 ± 0.7	97.7 ± 27.5	178.3 ± 33.0	24.6 ± 2.7
Total	80	52	43.9 ± 14.7	117.2 ± 11.1	71.0 ± 8.4	2.2 ± 0.6	102.6 ± 26.5	185.1 ± 31.7	24.5 ± 2.7

Data are presented as mean ± standard deviation.

### 2.3 Anthropometrics

Height without shoes was measured using a scale-mounted stadiometer to the nearest 0.5 cm, and weight was assessed using a digital scale (Health o Meter® Professional 500 KL, Sunbeam Products, Inc.). Body mass index (BMI) was subsequently calculated utilizing the standard formula weight in kilograms divided by height in meters squared.

### 2.4 Blood pressure

Following 10 min of supine rest, brachial and aortic blood pressure (BP) were measured in the nondominant arm (SphygmoCor XCEL, AtCor Medical, Inc.). The mean value of three BP measurements was used in analyses. Brachial pulse pressure was calculated as the difference between mean systolic and diastolic BP.

### 2.5 Vascular endothelial function via EndoPAT

Digital artery endothelium-dependent vasodilation was assessed via peripheral arterial tonometry (EndoPAT2000, Itamar Medical, Ltd.) as previously described (McCrea et al., 2012; Brant et al., 2013) and in accordance with the conditions specified by the manufacturer. Prior to use, the EndoPAT machine was turned on for 20 min. Following 10 min of supine rest, pneumatic finger-tip probes were placed on the index finger of each arm, and a blood pressure cuff was placed on the experimental (non-dominant) upper arm, whereas the other arm served as the contralateral control, with both arms at rest on arm supports. After a baseline recording of pulse amplitude for 5 min on each arm for equilibration, the blood pressure cuff on the experimental arm was inflated to 200 mmHg or 60 mmHg higher than the participant’s systolic blood pressure (whichever was higher) for 5 min to occlude the brachial artery. The cuff was then deflated to induce reactive hyperemia and post-occlusion peripheral arterial tonometry (PAT)-signals were recorded for an additional 5 min in both arms.

Reactive hyperemia index (RHI), an index of flow-mediated dilation, was derived as the ratio of the average pulse wave amplitude during hyperemia (60 to 120 s of the post-occlusion period) to the average pulse wave amplitude during baseline in the occluded hand, divided by the same value in the control hand and then multiplied by a baseline correction factor.

### 2.6 Lipid panels

Following measurements of vascular endothelial function, hemodynamics, and arterial stiffness, blood samples (100 μL) were collected from the antecubital vein in a lithium heparin tube. Samples were immediately analyzed for total cholesterol (TC), high-density lipoprotein cholesterol (HDL), triglycerides (TRIGs), non-high-density lipoprotein cholesterol (non-HDL), total cholesterol/HDL ratio (TC/HDL), low-density lipoprotein cholesterol (LDL), and very low-density lipoprotein cholesterol (VLDL) using a Piccolo Metlyte Plus CRP Reagent Disc on the Piccolo Xpress Chemistry Blood Analyzer (Abaxis, Union City, CA, USA).

### 2.7 Stool sample collection and processing

In all studies, stool samples were collected at home, stored refrigerated, and brought into the clinic within 24 h. In the clinic, they were stored at 4 °C and processed within 24 h in the following manner: two sterile cotton swabs were inserted into the stool at three separate locations and then stored at −80 °C for DNA extraction. Fecal DNA extraction was completed using the FastDNA^®^ Kit (MP Biomedicals, #116540400) per manufacturer’s protocol. Amplification of the V4 region of the 16S rRNA gene was carried out utilizing following the Earth Microbiome Project protocol using the 515F-806R primer set (Caporaso et al., 2012) containing a unique 12 bp error-correcting barcode included on the forward primer. Cycling and sequencing conditions were as previously described (Lee et al., 2020). DNA extraction controls, no template PCR controls, and the Zymo mock community were included on each sequencing plate.

### 2.8 Data processing and statistical analysis

Sequence reads were imported into QIIME2 (version 2024.2) for quality control (Bolyen et al., 2019). Briefly, the sequence reads were demultiplexed and concatenated. Utilizing a Phred score cutoff of 30 in all three studies, sequences were examined for quality filtering. Upon examining the demultiplexed data, sequences from all three studies were trimmed to 199 base pairs for the forward reads and 149 base pairs for the reverse reads. All reads were binned into amplicon sequence variants (ASVs) using the DADA2 paired-end pipeline (Callahan et al., 2016). Following denoising, the resulting ASV tables and representative sequences from all three studies were merged, and taxonomic assignments were made using Silva version 138. Samples with low reads or suspected contamination were removed, and mitochondrial and chloroplast sequences were filtered from the remaining samples.

The resulting ASV abundance table and metadata were imported into R (version 4.4.2) for secondary workflow. MicrobiomeAnalystR (version 2.0) marker data profiling was used for filtering, normalization, and global diversity analysis (Chong et al., 2020; Lu et al., 2023). Briefly, the minimum count filter was set at 4, and the low count filter was set at 10% based on prevalence in samples. Data normalization was performed based on total sum scaling. Alpha diversity was examined utilizing the Shannon diversity index and Chao1 index at the phyla and genus taxonomic levels, and comparisons were analyzed using nonparametric Mann–Whitney/Kruskal-Wallis tests. Beta diversity was calculated using the Principal Coordinates Analysis (PCoA) ordination method, with Bray-Curtis distances calculated at the phyla and genus taxonomic levels and analyzed using PERMDISP and PERMANOVA statistics. Statistical significance for alpha and beta diversity analyses was assigned as *p* < 0.05 and a false discovery rate <0.05.

It is well established that differential abundance tools commonly used in microbiome data analysis produce significantly different results and often overpredict differentially abundant taxa (Nearing et al., 2022; Yang and Chen, 2022). Thus, as proposed by Nearing and colleagues, we chose a consensus-based differential abundance approach to bolster our analyses and subsequent conclusions. Additionally, rather than collapsing by a higher taxonomic level, we examined differential abundance at the feature level. Unlike higher-level taxonomic comparisons, which collapse multiple taxa into a single identifier, feature-level analysis examines the abundance of each amplicon sequence variant (ASV) individually. This approach allowed us to investigate changes in the abundance of specific taxa that may drive CMD but would otherwise be obscured by grouping at higher taxonomic levels.

Filtered non-normalized ASV tables and corresponding metadata were extracted from the MicrobiomeAnalystR mbSet object for input into differential abundance tools ANCOMBC-2 (Lin and Peddada, 2020) (version 2.6.1), metagenomeSeq (Paulson et al., 2013) (version 1.46.0), corncob (Martin et al., 2020) (version 0.4.1), and DESeq2 (Love et al., 2014) (version 1.44.0). In all tools, differential abundance was analyzed using the formula *x + study + sex + age_cat*, where x equals the anthropometric or cardiometabolic parameter of interest. Statistical significance was assigned as *p* < 0.05 and a false discovery rate <0.1. This higher threshold was chosen to detect taxa that are changing and may be biologically important, even if they are not statistically significant, as it is well established that small shifts in taxa can profoundly impact host physiology. Taxa were identified as differentially abundant based on consensus, defined as taxa meeting the statistical significance threshold, identified as differentially abundant by ≥2 differential abundance tools, and in which the directionality of the fold change identified by each method agreed. The workflow utilized to run each differential abundance tool to determine consensus is described below.

#### 2.8.1 ANCOMBC-2

Using the phyloseq function from the phyloseq R package, filtered non-normalized ASV tables and metadata files were converted into a phyloseq object. As data were pre-filtered in MicrobiomeAnalystR, prevalence cutoff (prv_cut) and library cutoff (lib_cut) were set to zero. The group was set to the cardiometabolic or anthropometric parameter of interest, and the *p*-value adjustment (p_adj_method) set to Benjamini-Hochberg (“hochberg”). All other settings were default. Significant taxa were exported for each pairwise comparison for downstream identification of consensus taxa as described above.

#### 2.8.2 MetagenomeSeq

An MR experiment object was created using filtered ASV count data and corresponding metadata. Normalization was performed using the cumulative sum scaling (CSS) method implemented in the cumNorm function. Cardiometabolic and anthropometric parameters of interest were defined as factors with ordered levels, and pairwise comparisons were defined. Pairwise comparisons were generated and looped through using a custom R script (available here). For each pairwise comparison, data were subset to include relevant groups. Differential abundance testing was performed using the fitZig function, which fits a zero-inflated Gaussian model to normalized ASV counts to account for sparsity and overdispersion. *p*-values were adjusted for multiple comparisons using the Benjamini-Hochberg (“hochberg”) via the adjustMethod function. All other settings were default. Significant taxa were exported for each pairwise comparison for downstream identification of consensus taxa as described above.

#### 2.8.3 Corncob

Using the phyloseq function from the phyloseq R package, filtered non-normalized ASV tables and metadata files were converted into a phyloseq object. Each cardiometabolic and anthropometric parameter of interest was set as a factor with ordered levels. Pairwise comparisons were looped through using a custom R script (available here) as follows. For each comparison, filtered, non-normalized ASV tables and corresponding metadata were subset for each pairwise comparison. Differential abundance testing was performed using the differentialTest function, which uses a beta-binomial model to account for sparsity and overdispersion. To identify taxa that differed only in abundance, the null overdispersion model was set as identical as the non-null model. Wald tests with default non-bootstrap setting were used for significance testing and the default Benjamini-Hochberg (“hochberg”) method for and *p*-value adjustment. Significant taxa were exported for each pairwise comparison for downstream identification of consensus taxa as described above.

#### 2.8.4 DESeq2

Each cardiometabolic and anthropometric parameter of interest was set as a factor with ordered levels. Pairwise comparisons were generated and looped through using a custom R script (available here) as follows. Filtered non-normalized ASV table and metadata were subset for each pairwise comparison, and a DESeq2 dataset (dds) was created using the DESeqDataSetFromMatrix function. Size factors were calculated using the “poscounts” method, which performs a modified relative log expression normalization to handle sparsity and overdispersion. Differential abundance testing was performed using the DESeq function. *p*-value adjustment was conducted using the default Benjamini-Hochberg (“hochberg”) method. Significant taxa were exported for each pairwise comparison for downstream identification of consensus taxa as described above.

### 2.9 Microbiome co-occurrence network analyses

Microbial co-occurrence networks were constructed using the microeco R package (Liu et al., 2020; Liu C. et al., 2025) (version 1.15.0). A microtable object was created using the filtered, non-normalized ASV table, taxonomy table, and corresponding metadata. The microtable object was cloned and subset for each cardiometabolic and anthropometric parameter. Each subgroup was tidied and transformed to relative abundance prior to network construction. To account for the compositionality of microbiome data, networks were built using the SparCC correlation method (Friedman and Alm, 2012) via the SpiecEasi algorithm (Kurtz et al., 2015). A default correlation coefficient threshold of 0.6 was applied, with a *p*-value cutoff of < 0.01. *p*-value adjustments were performed using the default FDR method. Modules were detected using the walktrap algorithm. Network .gexf files were exported into Gephi (Bastian et al., 2009) for network visualization. To verify that the resulting networks deviated significantly from random networks, 100 random networks were generated and compared with the empirical networks (Supplementary Table 3).

Comparative network analyses were conducted using the meconetcomp package (Liu et al., 2023) (version 0.6.1). The edge_tax_comp function was used to evaluate the distribution of positive and negative edges at the Phylum level and visualized this data with heatmaps. Cohesion was calculated using the cohesionclass function, and significance was assessed using Wilcoxon rank-sum tests or Kruskal–Wallis tests followed by Dunn’s post-hoc comparisons when applicable. Robustness was evaluated using the robustness function with both edge and node-removal strategies. The relationships between removal ratio, network efficiency, and network connectedness were analyzed using Spearman’s correlation.

Recent evidence has demonstrated that network analyses can detect significant differences with as few as 10 samples, and that prediction accuracy tends to plateau at approximately 20–30 samples (Agyapong et al., 2025). Consequently, groups comprising fewer than 10 samples were excluded from the network analyses.

## 3 Results

### 3.1 Participant characteristics

On average, the study population (*N* = 132; 80 female, 52 male; age = 43.9 ± 14.7) was normal weight (BMI 24.5 ± 2.7) and self-reported as healthy. Study participants were normotensive (mean Systolic BP 117.2 ± 11.1, Diastolic BP 71.0 ± 8.4), had desirable TC (185.1 ± 31.7), above optimal LDL (102.6 ± 26.5), and normal RHI function (2.2 ± 0.6, Table 1, Supplementary Table 1). The percentage of subjects classified via clinical gradation of physiological parameters further conferred the health of the overall population, with 96.3% of subjects classified in the normal to overweight range for BMI, 60.3% normotensive, 66.2% desirable TC, 50.8% normal LDL, and 77.4% presented with normal RHI (Table 2).

**Table 2:** Categorical distribution of physiological parameters.

Cardiometabolic/Anthropometric parameter	Aronia Berry	Microgreens	Phage 2	Total
BP				131
Normal	17 (77.3%)	10 (43.5%)	52 (60.5%)	79 (60.3%)
Elevated	1 (4.5%)	9 (39.1%)	18 (20.9%)	28 (21.4%)
Stage 1	4 (18.2%)	4 (17.4%)	11 (12.8%)	19 (14.5%)
Stage 2	-	-	5 (5.8%)	5 (3.8%)
RHI				106
RHI normal	16 (72.7%)	-	66 (78.6%)	82 (77.4%)
RHI dysfunction	6 (27.3%)	-	18 (21.4%)	24 (22.6%)
LDL				128
Normal	6 (27.3%)	6 (28.6%)	53 (62.4%)	65 (50.8%)
Above optimal	10 (45.5%)	12 (57.1%)	24 (28.2%)	46 (35.9%)
Borderline high	6 (27.3%)	3 (14.3%)	5 (5.9%)	14 (10.9%)
High	-	-	3 (3.5%)	3 (2.3%)
TC				130
Desirable	12 (54.5%)	9 (39.1%)	65 (76.5%)	86 (66.2%)
Borderline high	10 (45.5%)	14 (60.9%)	16 (18.8%)	40 (30.8%)
High	-	-	4 (4.7%)	4 (3.1%)
BMI				132
Normal	12 (54.5%)	16 (69.6%)	58 (66.7%)	86 (65.2%)
Overweight	10 (45.5%)	7 (30.4%)	24 (27.6%)	41 (31.1%)
Obese	-	-	5 (5.7%)	5 (3.8%)

BP, blood pressure; RHI, reactive hyperemia index; LDL, low density lipoprotein; TC, total cholesterol; BMI, body mass index. Data is presented as the percentage of the total N for each physiological parameter of interest in each study, and as one cohort (Total).

### 3.2 Gut microbiota diversity differs by study, sex, and age, but not CMD parameters

**Figure 1:**
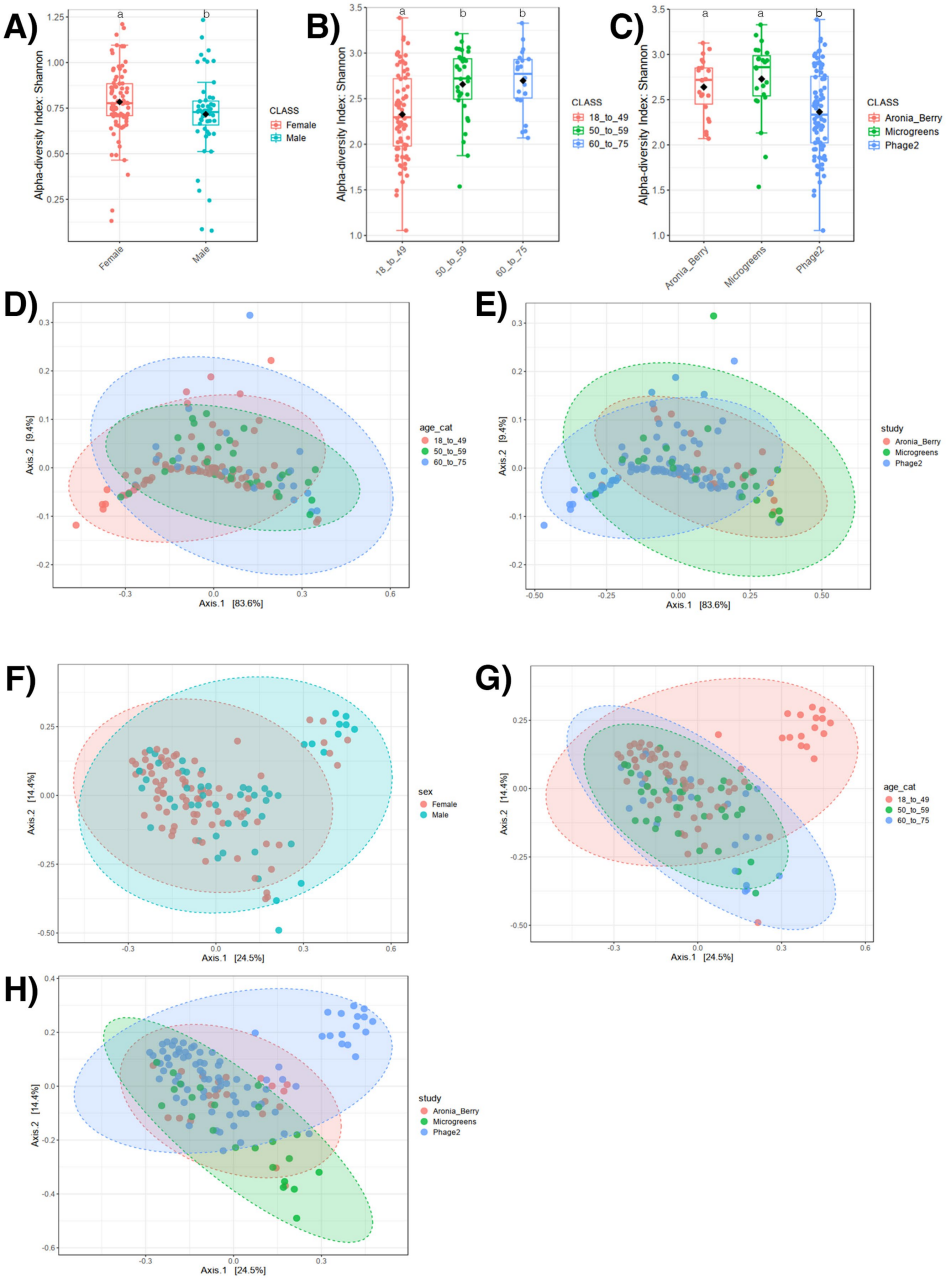
Box plots of Shannon’s diversity revealed statistically significant differences in phyla-level alpha diversity for sex **(A)** and genus-level alpha diversity for age **(B)** and study **(C)**. PCoA plots of Bray-Curtis diversity revealed statistically significant differences in phylum-level beta diversity for age **(D)** and study **(E)**, and genus-level beta diversity for sex **(F)**, age **(G)**, and study **(H)**. Shannon’s diversity was analyzed utilizing nonparametric Mann–Whitney/Kruskal-Wallis statistical tests. Bray-Curtis distances were analyzed using PERMANOVA statistics. Statistical significance was assigned as *p* < 0.05 and a false discovery rate <0.05.

Previous research has demonstrated that compared to healthy individuals, those with CMD exhibit alterations in gut microbial diversity (Fei et al., 2019; Walker et al., 2021). Utilizing 16 s rRNA amplicon sequencing, we evaluated differences in gut microbiota community diversity. Cardiometabolic and anthropometric parameters were stratified based on clinical definitions. At the phyla-level, no significant differences in Shannon’s or Chao1 alpha diversity indices were observed within clinical stratifications of cardiometabolic and anthropometric parameters of interest (Supplementary Figure 1). However, significant differences in phyla-level Shannon’s diversity index were identified between sexes (Shannon’s p.adj: <0.04, Figure 1A), with females having higher diversity compared to males.

**Figure 2:**
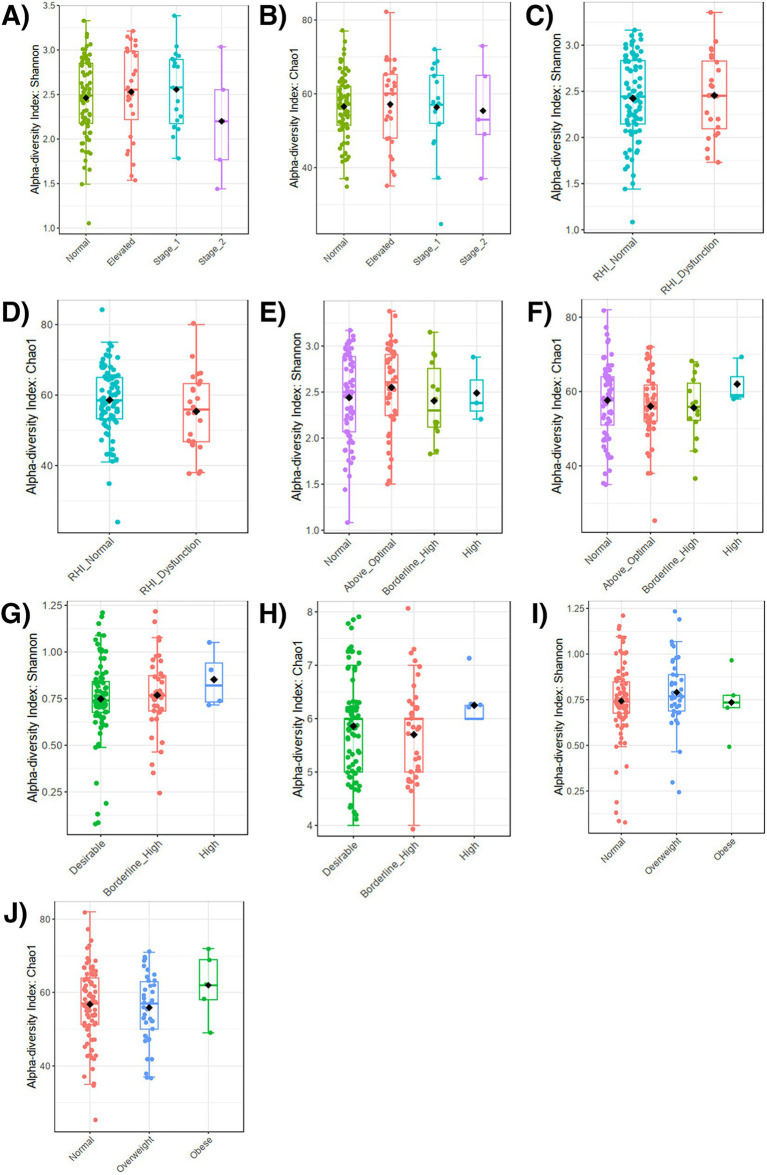
Box plots of genus level Shannon’s and Chao1 diversity indices for clinical stratifications of BP **(A,B)**, RHI **(C,D)**, LDL **(E,F)**, TC **(G,H)**, and BMI **(I,J)** revealed no statistically significant differences in alpha diversity. Shannon’s diversity indices were analyzed utilizing nonparametric Mann–Whitney/Kruskal-Wallis statistical tests. Statistical significance was assigned as *p* < 0.05 and a false discovery rate <0.05. BP, blood pressure; RHI, reactive hyperemia index; LDL, low density lipoprotein; TC, total cholesterol; BMI, body mass index.

Examining genus-level alpha diversity, analyses revealed no significant differences within cardiometabolic and anthropometric parameters (Figure 2). Significant genus-level differences were identified between age groups (Shannon’s *p*-value: <0.001, Figure 1B) and between studies (Shannon’s p-value: <0.001, Figure 1C). Compared to the 18 to 49 age group, both the 50 to 59 age group (Shannon’s p.adj: <0.001, Figure 1B) and the 60 to 75 age group (Shannon’s *p*-adj: <0.002, Figure 1B) displayed higher Shannon’s diversity. However, no significant differences were identified in the 50 to 59 age group compared to the 60 to 75 age group (Shannon’s p.adj: 0.91; Figure 1B). Additionally, compared to the Phage2 study, both the Aronia Berry (Shannon’s p.adj: <0.02, Figure 1C) and Microgreens studies (Shannon’s p.adj: <0.002, Figure 1C) had higher Shannon’s diversity indices. However, there were no significant differences between the Aronia Berry and Microgreens studies (Shannon’s p.adj: 0.22; Figure 1C).

Analysis of phyla-level beta-diversity (Supplementary Figure 2) utilizing Bray-Curtis distances visualized by PCoA did not demonstrate significant clustering by cardiometabolic and anthropometric parameters of interest. Significant differences in phyla-level Bray-Curtis diversity index were identified between age groups (PERMANOVA p.adj: 0.001, Figure 1D) and between studies (PERMANOVA p.adj: 0.001, Figure 1E). Pairwise analyses revealed significant differences in the 50 to 59 age group compared to the 18 to 49 age group (PERMANOVA p.adj: <0.002, Figure 1D), and the 60 to 75 age group compared to the 18 to 49 age group (PERMANOVA p.adj: <0.002, Figure 1D), but not between the 50 to 59 age group and 60 to 75 age group (PERMANOVA p.adj: 0.50; Figure 1D). Additionally, pairwise analyses showed significant differences in the Aronia Berry study compared to the Phage2 study (p.adj: <0.02, Figure 1E), and the Microgreens study compared to the Phage2 study (p.adj: <0.002, Figure 1E), but not between the Aronia Berry Study compared to the Microgreens study (p.adj: 0.26; Figure 1E).

**Figure 3:**
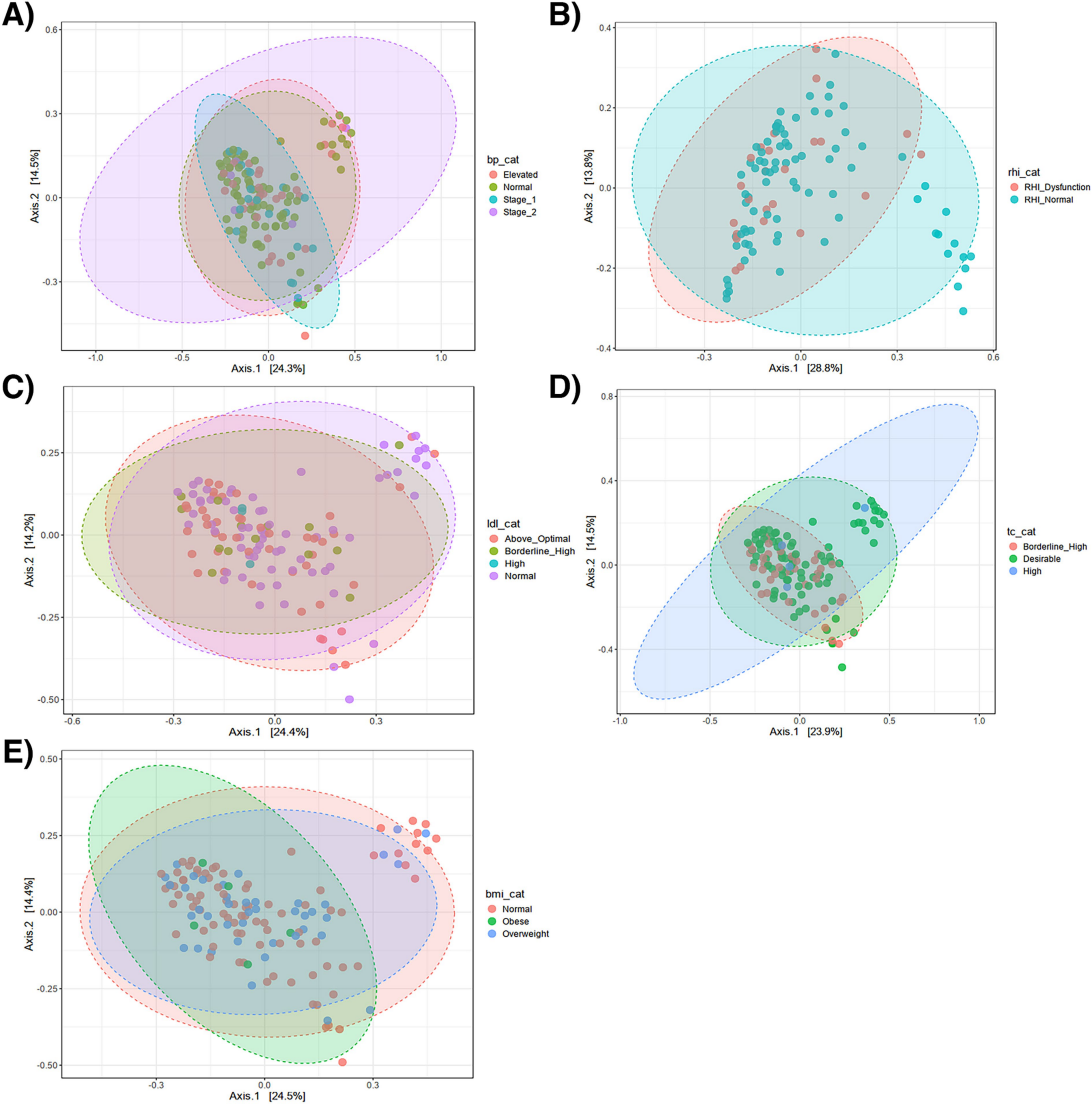
PCoA of genus level Bray-Curtis distances showed no significant separation of the gut microbiota across clinical stratifications of BP **(A)**, RHI **(B)**, LDL **(C)**, TC **(D)**, or BMI **(E)**, as calculated by PERMANOVA. Statistical significance was assigned as p < 0.05 and a false discovery rate <0.05. BP, blood pressure; RHI, reactive hyperemia index; LDL, low-density lipoprotein; TC, total cholesterol; BMI, body mass index.

Genus-level beta-diversity analyses (Figure 3) demonstrated a similar pattern, with no significant clustering by cardiometabolic or anthropometric parameter. Similarly, significant differences in genus-level Bray-Curtis diversity index were identified between sexes (*p*-value: 0.003, Figure 1F), between studies (PERMANOVA *p*-value: 0.001, Figure 1G), and with additional differences noted between age groups (PERMANOVA p-value: 0.002, Figure 1H). Pairwise analyses identified significant differences in the 50 to 59 age group compared to the 18 to 49 age group (PERMANOVA p.adj: <0.005, Figure 1H), and the 60 to 75 age group compared to the 18 to 49 age group (PERMANOVA p.adj: 003, Figure 1H), but not between the 50 to 59 age group and 60 to 75 age group (PERMANOVA p.adj: 0.39; Figure 1H). Additionally, pairwise analyses showed significant differences in the Aronia Berry study compared to the Phage2 study (p.adj: <0.002, Figure 1G), and the Microgreens study compared to the Phage2 study (PERMANOVA p.adj: <0.002, Figure 1G), as well as the Aronia Berry Study compared to the Microgreens study (PERMANOVA p.adj: <0.002; Figure 1G). Overall, these data suggest that there were no global compositional differences between clinical stratifications of cardiometabolic and anthropometric parameters of interest.

### 3.3 Gut microbiota differential abundance

Compared to healthy controls, individuals with abnormal cardiometabolic parameters frequently display differences in the abundance of individual taxa (Fei et al., 2019; Kim et al., 2018; Morales et al., 2022). Accordingly, we performed differential abundance analyses using ANCOMBC-II, metagenomeSeq, corncob, and DESeq2. As recommended by Nearing and colleagues (Nearing et al., 2022), we used a consensus-based approach to help ensure the robustness of interpretations.

**Figure 4:**
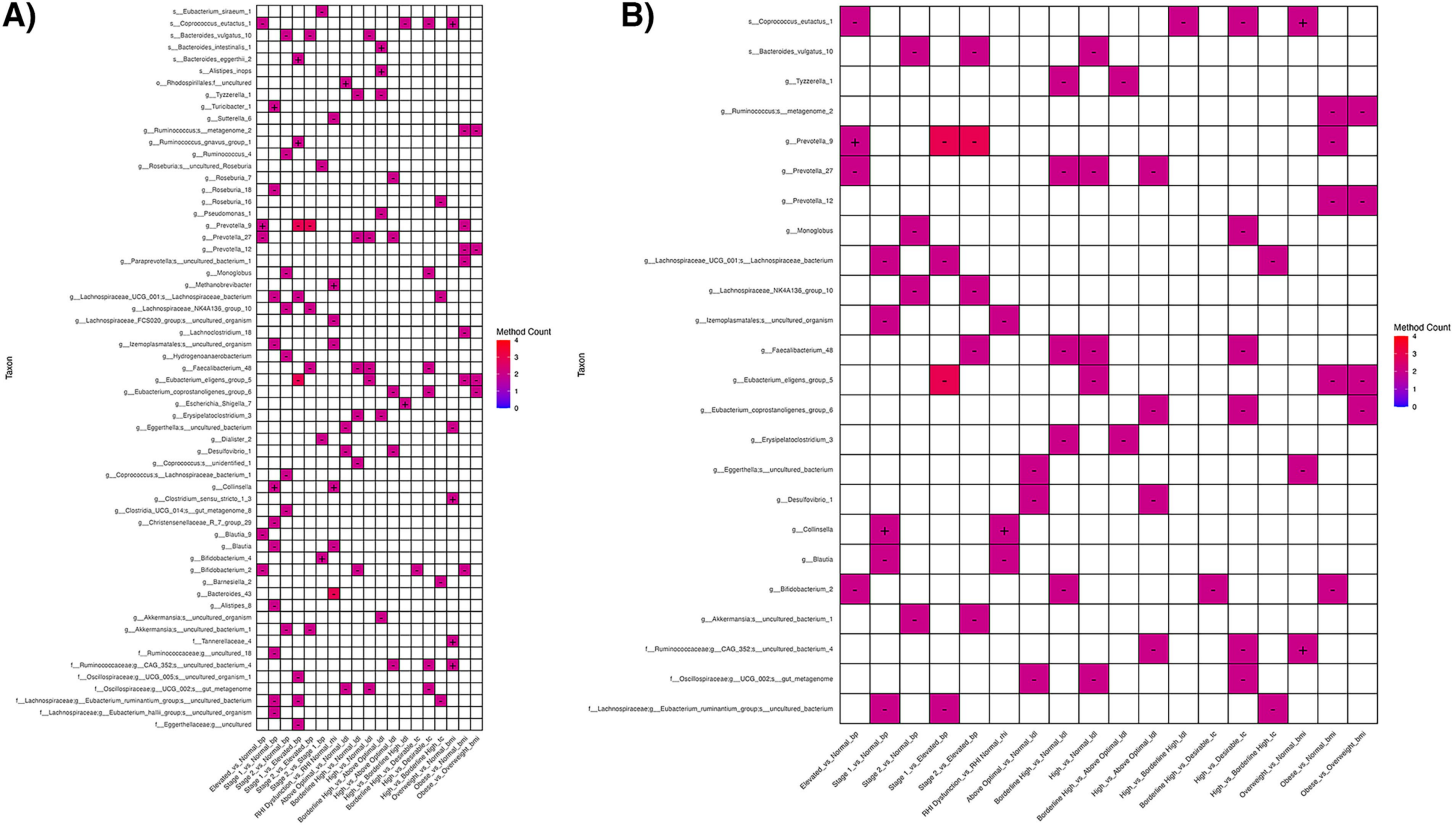
Heatmaps of all differentially abundant taxa **(A)**, and differentially abundant taxa identified in ≥2 comparisons **(B)**, at the ASV level for cardiometabolic parameters. Statistical significance was assigned as p < 0.05 and a false discovery rate <0.1. Direction of fold change is indicated by – or +. BP, blood pressure; RHI, reactive hyperemia index; LDL, low-density lipoprotein; TC, total cholesterol; BMI, body mass index.

After adjusting for sex, study, and age, differential abundance analyses identified several ASVs associated with clinical stratifications of cardiometabolic and anthropometric parameters. In total, 64 ASVs were found to be significantly differentially abundant across 103 comparisons (Figure 4A, Supplementary Table 2), with 16 comparisons showing increased abundance and 87 comparisons showing decreased abundance, when comparing levels of clinical stratifications. We then prioritized features identified in two or more clinical comparisons, resulting in 24 ASVs across 66 comparisons (Figure 4B, Supplementary Table 2), of which, 5 increased and 61 decreased in abundance, when comparing levels of clinical stratifications. These findings suggest that, compared with healthy controls, changes in cardiometabolic health are characterized by decreases in specific microbial taxa.

To determine whether changes in ASV abundance are associated with cardiometabolic and anthropometric parameters, Spearman correlation analyses were performed on features identified as differentially abundant across ≥2 clinical comparisons. Multiple significant associations were observed; however, none remained significant after adjustment for multiple comparisons (Supplementary Figure 3). Furthermore, all correlation coefficients were negligible to weak, with the strongest positive and negative correlations being +0.26 and −0.21, respectively. These findings suggest that shifts in the abundance of individual ASVs are unlikely to be major drivers of CMD progression.

### 3.4 Network analyses revealed shifts in microbial co-occurrence patterns

**Figure 5:**
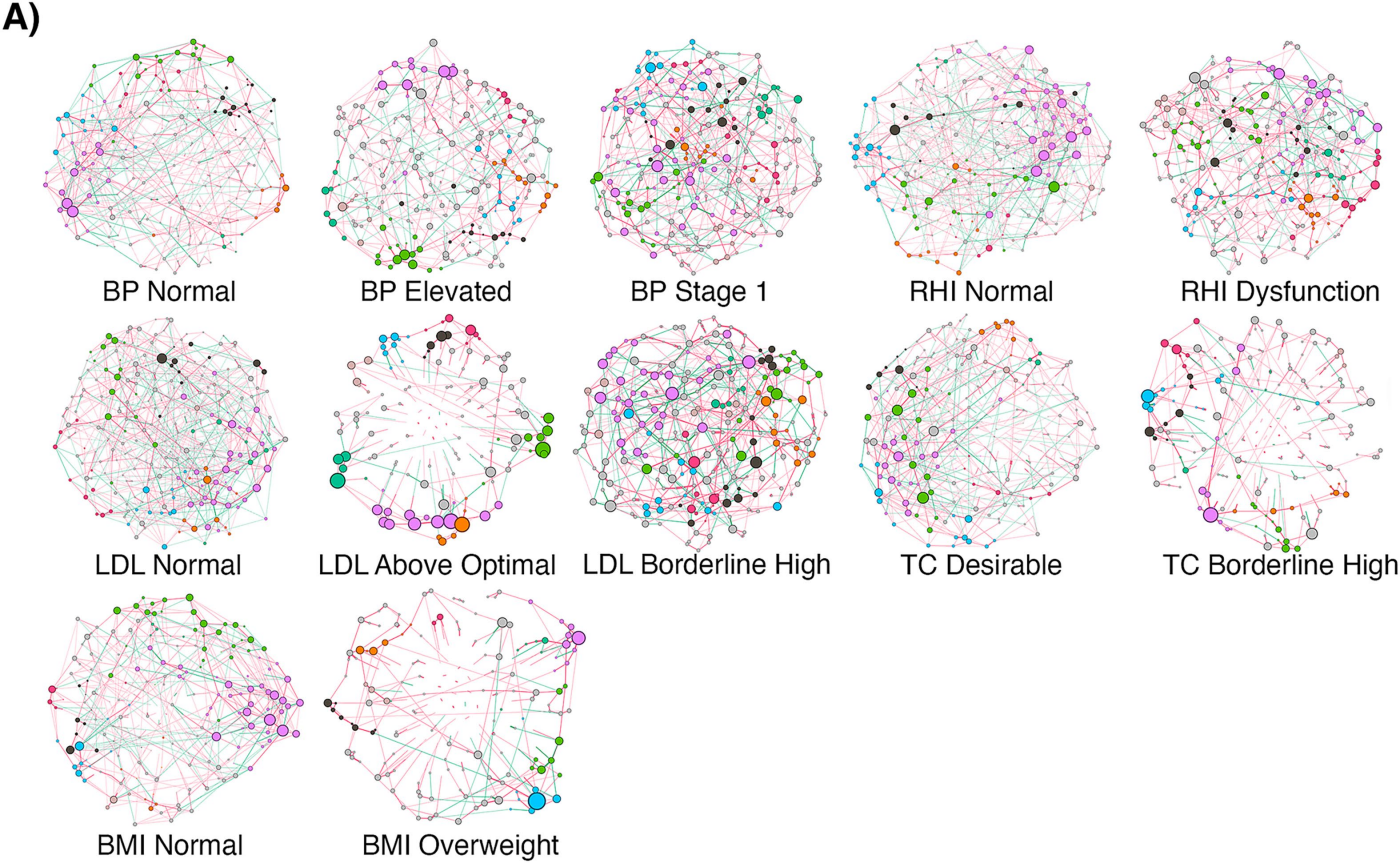
Co-occurrence networks of stratifications in cardiometabolic and anthropometric parameters. Color of nodes denotes modules in each network. Line color denotes positive (green) and negative (red) correlation coefficients. Networks were constructed using the SpiecEasi network method with SPARCC correlations, using a correlation coefficient threshold of 0.6, a *p*-value cutoff of 0.01, and FDR *p*-value adjustment. BP, blood pressure; RHI, reactive hyperemia index; LDL, low density lipoprotein; TC, total cholesterol; BMI, body mass index.

Studies have shown that individuals with CMD display alterations in gut microbiome 16S co-occurrence network parameters compared to healthy controls. In a cohort of individuals with obesity, Chen and colleagues identified shifts in microbial co-abundances associated with atherosclerosis and obesity (Chen et al., 2020). Xia et al. **(**2024**)** found that elderly individuals with hyperlipidemia exhibited markers of reduced gut microbial network stability compared with healthy controls. Consequently, we built co-occurrence networks based on significant correlations among ASVs (correlation coefficient <0.6; *p*-value < 0.01) (Figure 5). Network characteristics differed across stratifications of cardiometabolic and anthropometric parameters (Figure 5, Table 3).

**Table 3:** Characteristics of co-occurrence networks across clinical stratifications of cardiometabolic and anthropometric parameters.

Cardiometabolic/Anthropometric parameter	Vertex	Edge	Average degree	Average path length	Clustering coefficient	Density	Heterogeneity	Centralization	Modularity
BP									
Normal	274	523	3.82	0.09	0.09	0.01	0.64	0.05	0.79
Elevated	281	445	3.17	0.19	0.06	0.01	0.49	0.02	0.86
Stage 1	277	545	3.94	0.18	0.06	0.01	0.39	0.02	0.82
RHI									
Normal	289	632	4.37	0.07	0.08	0.02	0.64	0.04	0.74
Dysfunction	293	538	3.67	0.18	0.07	0.01	0.46	0.02	0.82
LDL									
Normal	287	687	4.79	0.06	0.07	0.02	0.50	0.04	0.74
Above Optimal	203	229	2.26	0.34	0.10	0.01	0.65	0.02	0.89
Borderline High	275	524	3.81	0.29	0.06	0.01	0.42	0.02	0.82
TC									
Desirable	275	505	3.67	0.10	0.08	0.01	0.67	0.04	0.80
Borderline High	257	285	2.22	0.37	0.09	0.01	0.61	0.03	0.91
BMI									
Normal	264	487	3.69	0.10	0.10	0.01	0.66	0.04	0.77
Overweight	224	246	2.20	0.35	0.08	0.01	0.61	0.03	0.89

BP, blood pressure; RHI, reactive hyperemia index; LDL, low-density lipoprotein; TC, total cholesterol; BMI, body mass index.

Network clustering coefficient is an indicator of how connected a node’s neighbors are to one another (Kajihara and Hynson, 2024). In general, networks of normal cardiometabolic and anthropometric measures showed higher clustering coefficients than networks of elevated measures (Table 3). The exceptions were above-optimal LDL (0.10) and borderline-high TC (0.09), for which the clustering coefficients were higher than those with normal blood lipids (Normal LDL 0.07; Desirable TC 0.08) (Table 3). Higher clustering coefficients may indicate cross-feeding relationships and network stabilization through efficient resource transfer (Lewinsohn et al., 2006; Wang et al., 2022). Average path length, which is the mean shortest path between all node pairs, serves as a measure of network efficiency (Kajihara and Hynson, 2024). Average path lengths were lower in networks constructed from microbiomes of individuals with normal cardiometabolic and anthropometric measures, suggesting more compact, efficient networks than those with elevated cardiometabolic parameters (Table 3). Modularity measures how a network is divided into subcommunities with higher internal connectivity than connections to the rest of the network (Kajihara and Hynson, 2024). All networks displayed high modularity (>0.70) (Table 3). Intriguingly, networks constructed from the gut microbiota of individuals with elevated cardiometabolic risk displayed higher modularity than those of healthy controls. Degree is a measure of the number of edges attached to a node (Kajihara and Hynson, 2024). Though a lower average degree has been associated with network stability (Wu et al., 2021), higher connectivity has also been postulated to be stabilizing as the network can compensate for the loss of nodes and edges in the face of disturbance (Guo et al., 2022). Apart from Stage 1 hypertension, networks derived from microbiomes of individuals with normal cardiometabolic parameters showed a higher average degree (Table 3). Together, these metrics indicate that the gut microbiomes of individuals with normal cardiometabolic parameters are more connected and have more efficient microbial interactions. In contrast, networks from individuals with elevated cardiometabolic traits are less connected and fragment into more modular structures.

**Figure 6:**
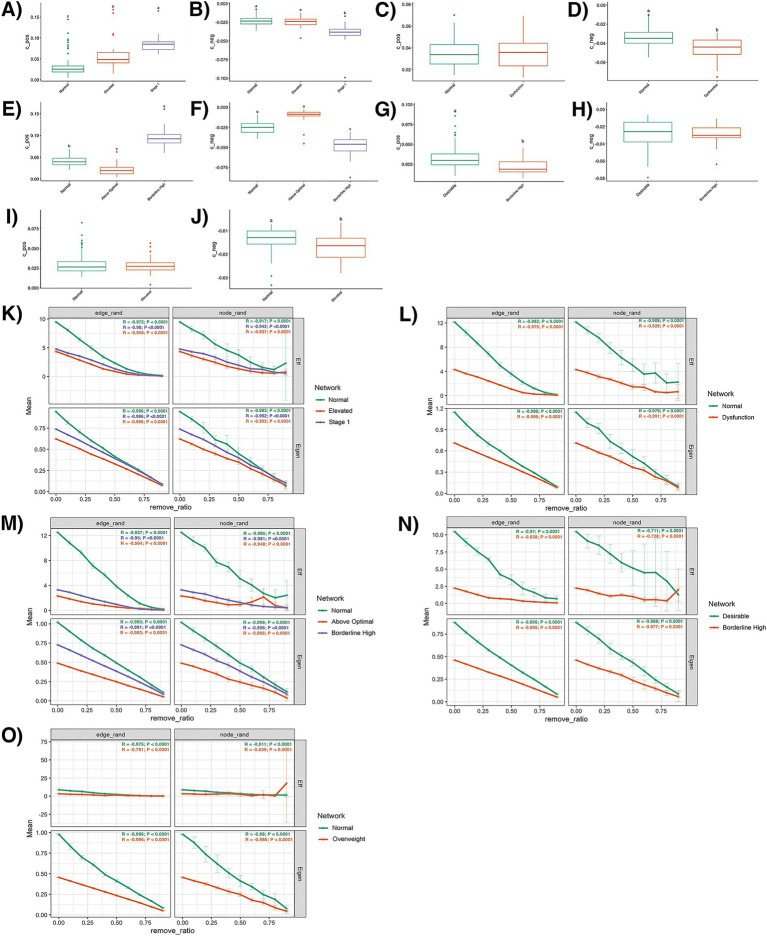
Positive and negative cohesion analyses revealed significant differences in microbial network connectivity for BP **(A,B)**, RHI **(C,D)**, LDL **(E,F)**, TC **(G,H)**, and BMI **(I,J)**. Robustness analyses revealed differences in microbial network ability to resist collapse upon targeted node and edge removal for BP **(K)**, RHI **(L)**, LDL **(M)**, TC **(N)**, and BMI **(O)**. BP, blood pressure; RHI, reactive hyperemia index; LDL, low density lipoprotein; TC, total cholesterol; BMI, body mass index.

Visualization of co-occurrence network connectivity revealed shifts in the ratio of positive and negative edges in those with elevated cardiometabolic parameters compared to healthy individuals (Supplementary Figures 4, 5). Cohesion calculates the abundance-weighted average of positive and negative correlations between taxa in a network (Herren and McMahon, 2017). Accordingly, we conducted cohesion analyses to quantify potential differences in positive and negative connections within these microbial communities (Figure 6). Analyses revealed that positive cohesion was variable across cardiometabolic parameters. Conversely, co-occurrence networks built from the microbiomes of individuals with normal cardiometabolic parameters displayed significantly lower negative cohesion than those built from individuals with elevated cardiometabolic risk.

Negative cohesion was significantly lower in normal compared to stage-1 hypertension (Normal BP −0.023 ± 0.006 vs. Stage 1-hypertension −0.04 ± 0.02; *p* < 0.001) and elevated BP vs. stage 1-hypertension (Elevated BP −0.024 ± 0.007 vs. Stage 1-hypertension −0.04 ± 0.02; *p* < 0.001), but not normal compared to elevated (Normal BP −0.023 ± 0.006 vs. Elevated BP −0.024 ± 0.007; *p* < 0.7; Figure 6B). Normal RHI networks displayed significantly lower negative cohesion compared to RHI dysfunction (Normal Function −0.033 ± 0.01 vs. Dysfunction −0.046 ± 0.013; *p* < 0.001) (Figure 6D). Examining LDL networks, negative cohesion was significantly lower in normal compared to borderline high (Normal LDL −0.025 ± 0.007 vs. Borderline High LDL −0.05 ± 0.014; *p* < 0.001) and in above optimal compared to borderline high (Above Optimal LDL −0.01 ± 0.007 vs. Borderline High LDL −0.05 ± 0.014; *p* < 0.001; Figure 6F). However, negative cohesion was significantly lower in above optimal compared to normal LDL (Normal −0.025 ± 0.007 vs. Above Optimal −0.01 ± 0.007; *p* < 0.001; Figure 6F), TC displayed no significant differences in negative cohesion between groups (Desirable TC −0.025 ± 0.007 vs. Borderline High TC −0.01 ± 0.007; *p* < 0.001) (Figure 6H). Finally, negative cohesion was significantly lower in normal than in overweight networks (Normal weight: −0.014 ± 0.005 vs. Overweight: −0.017 ± 0.006; *p* < 0.001) (Figure 6J). These findings indicate that as CMD progresses, microbial networks exhibit increasingly intense competitive interactions.

Next, we evaluated network robustness—the capacity to withstand network collapse in the presence of disturbances (Kajihara and Hynson, 2024). Network disturbances were computationally imposed by randomly removing edges and nodes. Networks built from the microbiota of individuals with normal cardiometabolic parameters exhibited higher efficiency and eigenvector centrality than those from individuals with elevated cardiometabolic parameters, both before and after random removal of edges and nodes (Figures 6K–O). Edge and node removal showed significant negative correlations with robustness across all groups. These results suggest that gut microbial networks in individuals with normal cardiometabolic health are more resistant to network disturbances than in those with elevated CMD risk.

## 4 Discussion

Over the last two decades, gut microbiota has emerged as a key player in cardiometabolic health and CMD development (Battson et al., 2018b; Tang and Hazen, 2024). Compared with healthy controls, individuals with overt CMD tend to exhibit shifts in gut microbial diversity indices, such as reduced alpha diversity and/or differences in beta diversity (Lopez-Montoya et al., 2022; Pinart et al., 2021; Sun S. et al., 2019; Louca et al., 2021; Fei et al., 2019). However, when stratifying by clinical stratifications of CMDs, we found no differences in alpha or beta diversity across cardiometabolic variables of interest. This was observed even when comparing individuals with overt disease, such as stage 2 hypertension, high LDL or TC, and obesity, to individuals with normal clinical biomarkers. While this latter finding contradicts much of the literature examining differences in gut microbial diversity in individuals with overt hypertension, dyslipidemia, and obesity (Lopez-Montoya et al., 2022; Pinart et al., 2021; Sun S. et al., 2019; Louca et al., 2021; Fei et al., 2019), we postulate this is due to the low number of study participants with overt CMD in our study cohorts. Accordingly, comparisons involving overt disease in this combined clinical dataset are exploratory and should be interpreted cautiously. Although no differences were identified among the parameters of interest, we did observe significant differences in alpha and beta diversity indices by sex and age. Specifically, females exhibited higher Shannon’s diversity at the phylum level relative to males, and older individuals demonstrated greater genus-level alpha diversity compared to their younger counterparts. These findings largely align with the existing literature, with females frequently exhibiting higher alpha diversity metrics than males (de la Cuesta-Zuluaga et al., 2019; Gao et al., 2018; Ghaffar et al., 2025; Sinha et al., 2019), particularly among younger adults, while older individuals display higher alpha diversity compared to their younger counterparts (Peters et al., 2025). However, the results are heterogeneous, with differences in diversity comparisons varying across studies for both sex (de la Cuesta-Zuluaga et al., 2019; Ghaffar et al., 2025) and age (Wilmanski et al., 2021). This variability may be attributed to numerous factors, including differences in study design, such as primers employed and sequencing and analysis approaches, as well as variables such as diet, lifestyle such as physical activity, and environment. Collectively, these data indicate that large-scale compositional shifts, characterized by differences in alpha and beta diversity when compared to healthy controls, may be a consequence rather than a cause of CMD. Instead, specific pathobionts and the changes they induce in the microbial community may drive disease progression.

The driver-passenger hypothesis proposes that highly pathogenic bacteria (drivers) may initiate disease processes and induce changes in the gut microbial community through modification of the host ecosystem. Bacteria better adapted to the early disease ecosystem (passengers) can outcompete others, including the drivers, for resources. As a result, by the time major compositional changes are seen in overt disease, it may be too late to identify the original driver bacteria or the subtle microbial changes that triggered disease progression. Some research indicates that drivers may trigger vascular dysfunction and host metabolic changes, highlighting the plausibility of the driver-passenger hypothesis in CMD progression. For instance, in gnotobiotic mice colonized with a synthetic community of human gut microbiome commensals unable to produce trimethylamine (TMA), transplantation of *Clostridium sporogenes*, which harbors the choline utilization C gene (CutC) responsible for the metabolism of choline into TMA, resulted in enhanced platelet responsiveness, a faster rate of thrombus formation, and a reduced time to vessel occlusion (Skye et al., 2018). Remarkably, transplanted *C. sporogenes* only comprised ~0.1% of the microbiome. Similarly, in a model of carotid injury, transplantation of *Bacteroides thetaiotaomicron*, which synthesizes L-tryptophan, was shown to increase platelet reactivity, accelerate thrombus formation, and shorten the time to vessel occlusion in germ-free ApoE −/− mice (Liu et al., 2024). As previously discussed, the transplantation of *M. rupellensis* into germ-free mice facilitated obesity by increasing lipid absorption through myo-inositol degradation (Wu et al., 2024). Collectively, these findings suggest that individual bacterial species may drive early progression of vascular and metabolic dysfunctions characteristic of CMD.

To identify drivers, we conducted an ASV-level consensus-based differential abundance analysis. This systematic approach enhanced the biological plausibility of the identified taxa by reducing false positives commonly identified with a single method and enabled us to analyze changes in the abundance of specific taxa that may influence CMD but would be obscured when grouped at higher taxonomic levels. After adjusting for sex, study, and age, consensus analysis identified 64 features that were significantly differentially abundant across stratifications of cardiometabolic parameters. Of those, 24 features were identified as differentially abundant in ≥2 clinical comparisons. However, no features were identified as differentially abundant across all forms of CMD. Furthermore, while Spearman correlation analyses identified several significant associations between several ASVs and cardiometabolic parameters, none remained significant after correction for multiple comparisons, and all correlation coefficients were negligible to weak. These findings indicate that individual ASVs are unlikely to represent drivers of CMD progression in this cohort. Nevertheless, differential abundance analysis revealed a pattern that may be important in the progression of CMD.

Interestingly, with few exceptions, most taxa identified as differentially abundant were decreased as cardiometabolic risk factors increased. As discussed in detail in previous reviews (Fusco et al., 2023; Nogal et al., 2021; Kim et al., 2022), many of the taxa we identified as decreased in abundance, including *Akkermansia, Bacteroides, Bifidobacterium, Blautia, Eubacterium, Faecalibacterium, Lachnospiraceae, Oscillospiraceae, Prevotella, Roseburia,* and *Ruminococcus,* produce short-chain fatty acids (SCFAs), metabolites shown to play a key role in cardiometabolic health. For instance, SCFAs regulate vascular function and blood pressure (Natarajan et al., 2016; Pluznick, 2014; Pluznick et al., 2013) and modulate blood lipid levels (Zheng et al., 2024). SCFAs also play a key role in immune function and have been shown to modulate repair post myocardial infarction (Tang et al., 2019). These data also align with our previous findings in murine models, in which we observed that vascular dysfunction was associated with the loss of beneficial taxa rather than the presence of specific pathogenic organisms (Battson et al., 2019). In humans, the recent ZOE Microbiome Health Ranking 2025 found that health-associated bacteria were present at higher levels in control subjects than in cases across 21 of 25 case–control validation cohorts (Asnicar et al., 2025). Together, these data suggest that rather than specific drivers of CMD, the loss of health-associated bacteria may underlie CMD progression.

In addition to the loss of health-associated taxa, the characteristics of co-occurrence networks varied across different stratifications of cardiometabolic parameters. Co-occurrence networks of individuals with elevated cardiometabolic parameters displayed a higher average path length, higher modularity, higher negative cohesion, lower clustering coefficient, lower average degree, and lower robustness than networks built from individuals with normal cardiometabolic parameters. Although network analyses are common in studies of environmental microbiomes like soil, network changes in the context of CMD have not been thoroughly explored. Ding et al. reported differences in co-occurrence networks of individuals with hypertension or hypertension and type 2 diabetes (T2D) compared to healthy controls (Ding et al., 2023). Contrary to our findings, healthy controls exhibited a higher average path length, suggesting reduced network efficiency, in comparison to individuals with hypertension and T2D, with no significant difference when compared to individuals with hypertension alone. These discrepancies may be attributed to differences in network construction methodologies or cohort variations, such as age and ethnicity. In line with the present findings, individuals with hypertension and T2D had a lower average degree than healthy controls, while, interestingly, individuals with hypertension alone had a higher average degree. This finding is consistent with our results, as individuals with normal cardiometabolic parameters exhibited a higher average degree, except when compared to individuals with stage 1-hypertension. In line with the driver-passenger hypothesis, this may suggest that changes in the host environment induced by overt hypertension, in turn, may alter the connectivity of the gut microbiota network. Additionally, these findings may suggest unique network shifts as a result of overt hypertension as compared to other CMDs. Ding and colleagues found that healthy controls had higher abundance of health-associated bacteria, including *Bacteroides, Bifidobacterium, Blautia, Faecalibacterium, Lachnospiraceae, and Prevotella.* Furthermore, as discussed above, Aniscar and team found that control subjects had higher levels of health-associated bacteria than cases in 21 of 25 validation cohorts (Asnicar et al., 2025). In light of our findings, this may support the hypothesis that the loss of health-associated bacteria contributes to the development of CMD. Additional research has demonstrated shifts in the co-abundance of taxa in metabolic syndrome (Fang et al., 2025), hypertension (Liu L. et al., 2025) obesity (Chen et al., 2020; Wang et al., 2021), and hyperlipidemia (Xia et al., 2024), but they did not report network metrics, thereby limiting direct comparisons to the present study and underscoring the need for further research to understand how changes in network structure may contribute to CMD progression.

Current understanding of microbial ecology suggests the identified network shifts may be explained by the loss of health-associated taxa identified via differential abundance analysis. Primary degraders and fermenters such as *Akkermansia, Bacteroides,* and *Bifidobacterium, Blautia, and Prevotella* produce public goods, such as acetate and lactate, which are cross-fed to other bacteria (Fernandez-Julia et al., 2022; Gao et al., 2025; Xiao et al., 2024; Culp and Goodman, 2023). The loss of these bacteria in individuals with elevated cardiometabolic parameters may be reflected in the lower clustering coefficient and a higher average path length. A higher clustering coefficient has been suggested to indicate increased cross-feeding relationships and more efficient resource transfer, while a lower average path length reflects increased network efficiency (Kajihara and Hynson, 2024). Furthermore, a reduction in the abundance or loss of primary degraders and fermenters may, in turn, reduce availability of public goods causing a decrease in secondary fermenters like *Lachnospiraceae* and *Oscillospiraceae* (Culp and Goodman, 2023), which we also identified as reduced in individuals with elevated cardiometabolic risk. The loss of these taxa may further decrease network connectivity (degree), increase competitive interactions (negative cohesion), and enhance network fragmentation (modularity). While networks from individuals with normal cardiometabolic parameters showed greater overall robustness, systematically removing nodes or edges caused similar proportional declines in network integrity across all groups. However, the decreased connectivity, increased fragmentation, and patterns that suggest increased competitive interactions identified in individuals with elevated cardiometabolic parameters may hinder the recovery of health-related bacteria. This could be associated with the gut microbiota remaining in an unhealthy state and, in turn, with the progression of CMD.

### 4.1 Limitations

Although the current study has several advantages that strengthen the associations identified, several limitations should be acknowledged. First, while we cannot entirely rule out residual confounding from variations in recruitment criteria, the inclusion of diverse cohorts may improve the applicability of our results. The persistence of microbiome associations across populations after accounting for study, age, and sex indicates that the loss of health-associated taxa may represent core elements of cardiometabolic disease biology. Second, the study does not include the quantification of microbial metabolites such as short-chain fatty acids (SCFAs). Although many of the taxa we identified as decreased in abundance are well-documented producers of SCFAs, quantification is necessary to determine whether individuals exhibiting elevated cardiometabolic risk have lower SCFA levels compared to those with normal cardiometabolic parameters. The small sample size, particularly those with overt CMD, and lack of analysis of mediating factors such as diet and exercise are also limitations to the research. Third, despite the robustness of the network analyses utilized, causality cannot be determined from these cross-sectional data. More research is required to elucidate the impact of changes in microbial co-occurrence networks and CMD progression. Finally, it is well established that the roles of gut microbes vary with the host’s geographic region and traits. Therefore, extrapolation of these results to broader CMD populations should be done with caution. Further research is needed to determine whether the observed patterns are consistent across individuals from different geographic locations, cultural backgrounds, dietary patterns, health conditions, etc.

## 5 Conclusion

In summary, the present study calls into question the role of global shifts in diversity as a driver of CMD. In individuals with CMD, including elevated BP and hypertension, vascular dysfunction, dyslipidemia, and high BMI, we found no differences in alpha or beta diversity compared to healthy controls. Importantly, these data may indicate that, rather than large-scale compositional changes, even modest shifts in the abundance of health-associated bacteria could be linked to the development of CMD. Through interactions with other gut bacteria and host-microbiome axes, reduced production of public goods and beneficial metabolites, such as SCFAs, may drive CMD onset. In turn, decreased network connectivity and increased network fragmentation may stabilize the gut microbiome in an unhealthy state, wherein the continued absence of health-related taxa and metabolites further promotes the meaningful progression of CMD. This underscores the importance of examining functional and network changes rather than taxonomic differences to better understand how the gut microbiota influences the development of CMD. As technology for analyzing the gut microbiome advances, it is imperative that microbiome research shifts from traditional association studies to the exploration of causal mechanisms of disease, which may lead to the discovery of gut microbial biomarkers for earlier detection of disease states and to therapeutic targets that prevent the onset of CMD.

## Data Availability

The 16S sequencing data are publicly available in the European Nucleotide Archive under the accession number of BioProject: PRJEB106625 (Phage2 Study), BioProject: PRJEB106718 (Microgreens Study), and BioProject: PRJEB106748 (Aronia Berry Study). The code used for data processing of the raw data, plus the code used to generate figures in this manuscript are available of GitHub at https://github.com/Intestinal-Health-Lab/Exploring-a-Theoretical-Framework-of-the-Gut-Microbiota-in-Cardiometabolic-Disease-Transition.

## References

[ref1] Agyapong D. Propster J. R. Marks J. Hocking T. D. (2025). Cross-validation for training and testing co-occurrence network inference algorithms. BMC Bioinformatics. 26:74. doi: 10.1186/s12859-025-06083-7, 40045231 PMC11883995

[ref2] Angell S. Y. Anderson C. A. M. Bibbins-Domingo K. Boyle D. S. Capewell S. Ezzati M. . (2020). The American Heart Association 2030 impact goal: a presidential advisory from the American Heart Association. Circulation 141, e120–e138. doi: 10.1161/CIR.0000000000000758, 31992057 PMC8690536

[ref3] Asnicar F. Manghi P. Fackelmann G. Baldanzi G. Bakker E. Ricci L. . (2025). Gut micro-organisms associated with health, nutrition and dietary interventions. Nature 650, 450–458. doi: 10.1038/s41586-025-09854-7, 41372407 PMC12893911

[ref4] Bansilal S. Castellano J. M. Fuster V. (2015). Global burden of CVD: focus on secondary prevention of cardiovascular disease. Int. J. Cardiol. 201, S1–S7. doi: 10.1016/S0167-5273(15)31026-3, 26747389

[ref5] Bastian M. Heymann S. Jacomy M. Gephi: An open source software for exploring and manipulating networks Proceedings of the International AAAI Conference on Web and Social Media (2009). doi: 10.1609/icwsm.v3i1.13937

[ref6] Battson M. L. Lee D. M. Jarrell D. K. Hou S. Ecton K. E. Weir T. L. . (2018a). Suppression of gut dysbiosis reverses Western diet-induced vascular dysfunction. Am. J. Physiol. Endocrinol. Metab. 314, E468–E477. doi: 10.1152/ajpendo.00187.2017, 29351482 PMC6048388

[ref7] Battson M. L. Lee D. M. Li Puma L. C. Ecton K. E. Thomas K. N. Febvre H. P. . (2019). Gut microbiota regulates cardiac ischemic tolerance and aortic stiffness in obesity. Am. J. Physiol. Heart Circ. Physiol. 317, H1210–H1220. doi: 10.1152/ajpheart.00346.2019, 31559829 PMC6960779

[ref8] Battson M. L. Lee D. M. Weir T. L. Gentile C. L. (2018b). The gut microbiota as a novel regulator of cardiovascular function and disease. J. Nutr. Biochem. 56, 1–15. doi: 10.1016/j.jnutbio.2017.12.010, 29427903

[ref9] Bolyen E. Rideout J. R. Dillon M. R. Bokulich N. A. Abnet C. C. Al-Ghalith G. A. . (2019). Reproducible, interactive, scalable and extensible microbiome data science using QIIME 2. Nat. Biotechnol. 37, 852–857. doi: 10.1038/s41587-019-0209-9, 31341288 PMC7015180

[ref10] Brant L. C. Barreto S. M. Passos V. M. Ribeiro A. L. (2013). Reproducibility of peripheral arterial tonometry for the assessment of endothelial function in adults. J. Hypertens. 31, 1984–1990. doi: 10.1097/HJH.0b013e328362d913, 23751970

[ref11] Callahan B. J. McMurdie P. J. Rosen M. J. Han A. W. Johnson A. J. Holmes S. P. (2016). DADA2: high-resolution sample inference from Illumina amplicon data. Nat. Methods 13, 581–583. doi: 10.1038/nmeth.3869, 27214047 PMC4927377

[ref12] Caporaso J. G. Lauber C. L. Walters W. A. Berg-Lyons D. Huntley J. Fierer N. . (2012). Ultra-high-throughput microbial community analysis on the Illumina HiSeq and MiSeq platforms. ISME J. 6, 1621–1624. doi: 10.1038/ismej.2012.8, 22402401 PMC3400413

[ref13] Chen L. Collij V. Jaeger M. van den Munckhof I. C. L. Vich Vila A. Kurilshikov A. . (2020). Gut microbial co-abundance networks show specificity in inflammatory bowel disease and obesity. Nat. Commun. 11:4018. doi: 10.1038/s41467-020-17840-y, 32782301 PMC7419557

[ref14] Chong J. Liu P. Zhou G. Xia J. (2020). Using MicrobiomeAnalyst for comprehensive statistical, functional, and meta-analysis of microbiome data. Nat. Protoc. 15, 799–821. doi: 10.1038/s41596-019-0264-1, 31942082

[ref15] Culp E. J. Goodman A. L. (2023). Cross-feeding in the gut microbiome: ecology and mechanisms. Cell Host Microbe 31, 485–499. doi: 10.1016/j.chom.2023.03.016, 37054671 PMC10125260

[ref16] de la Cuesta-Zuluaga J. Kelley S. T. Chen Y. Escobar J. S. Mueller N. T. Ley R. E. . (2019). Age- and sex-dependent patterns of gut microbial diversity in human adults. mSystems 4. doi: 10.1128/mSystems.00261-19, 31098397 PMC6517691

[ref17] Ding H. Xu Y. Cheng Y. Zhou H. Dong S. Wu J. . (2023). Gut microbiome profile of Chinese hypertension patients with and without type 2 diabetes mellitus. BMC Microbiol. 23:254. doi: 10.1186/s12866-023-02967-x, 37689641 PMC10492291

[ref18] Du Z. Qin Y. (2023). Dyslipidemia and cardiovascular disease: current knowledge, existing challenges, and new opportunities for management strategies. J. Clin. Med. 12. doi: 10.3390/jcm12010363, 36615163 PMC9820834

[ref19] Fang Y. Meng X. Cao R. Li J. Cai H. Liao P. . (2025). Alterations in gut microbial co-abundance networks in metabolic syndrome: a population-based cross-sectional study. Microorganisms 13. doi: 10.3390/microorganisms13122759, 41471963 PMC12736139

[ref20] Fei N. Bernabe B. P. Lie L. Baghdan D. Bedu-Addo K. Plange-Rhule J. . (2019). The human microbiota is associated with cardiometabolic risk across the epidemiologic transition. PLoS One 14:e0215262. doi: 10.1371/journal.pone.0215262, 31339887 PMC6656343

[ref21] Fernandez-Julia P. Commane D. M. van Sinderen D. Munoz-Munoz J. (2022). Cross-feeding interactions between human gut commensals belonging to the Bacteroides and Bifidobacterium genera when grown on dietary glycans. Microbiome Res Rep. 1:12. doi: 10.20517/mrr.2021.05, 38045648 PMC10688802

[ref22] Friedman J. Alm E. J. (2012). Inferring correlation networks from genomic survey data. PLoS Comput. Biol. 8:e1002687. doi: 10.1371/journal.pcbi.1002687, 23028285 PMC3447976

[ref23] Fuchs F. D. Whelton P. K. (2020). High blood pressure and cardiovascular disease. Hypertension 75, 285–292. doi: 10.1161/HYPERTENSIONAHA.119.14240, 31865786 PMC10243231

[ref24] Fusco W. Lorenzo M. B. Cintoni M. Porcari S. Rinninella E. Kaitsas F. . (2023). Short-chain fatty-acid-producing Bacteria: key components of the human gut microbiota. Nutrients 15. doi: 10.3390/nu15092211, 37432351 PMC10180739

[ref25] Gao F. Cheng C. Li R. Chen Z. Tang K. Du G. (2025). The role of *Akkermansia muciniphila* in maintaining health: a bibliometric study. Front. Med. 12:1484656. doi: 10.3389/fmed.2025.1484656, 39967592 PMC11833336

[ref26] Gao X. Zhang M. Xue J. Huang J. Zhuang R. Zhou X. . (2018). Body mass index differences in the gut microbiota are gender specific. Front. Microbiol. 9:1250. doi: 10.3389/fmicb.2018.01250, 29988340 PMC6023965

[ref27] Ghaffar T. Valeriani F. Romano Spica V. (2025). The sex related differences in health and disease: a systematic review of sex-specific gut microbiota and possible implications for microbial pathogenesis. Microb. Pathog. 209:108094. doi: 10.1016/j.micpath.2025.108094, 41062001

[ref28] Grubb D. S. Wrigley S. D. Freedman K. E. Wei Y. Vazquez A. R. Trotter R. E. . (2020). PHAGE-2 study: supplemental bacteriophages extend *Bifidobacterium animalis* subsp. *lactis* BL04 benefits on gut health and microbiota in healthy adults. Nutrients 12:2474. doi: 10.3390/nu12082474, 32824480 PMC7468981

[ref29] Guo Y. Song B. Li A. Wu Q. Huang H. Li N. . (2022). Higher pH is associated with enhanced co-occurrence network complexity, stability and nutrient cycling functions in the rice rhizosphere microbiome. Environ. Microbiol. 24, 6200–6219. doi: 10.1111/1462-2920.16185, 36076153

[ref30] Herren C. M. McMahon K. D. (2017). Cohesion: a method for quantifying the connectivity of microbial communities. ISME J. 11, 2426–2438. doi: 10.1038/ismej.2017.91, 28731477 PMC5649174

[ref31] Jones D. S. Greene J. A. (2012). The contributions of prevention and treatment to the decline in cardiovascular mortality: lessons from a forty-year debate. Health Aff. (Millwood). 31, 2250–2258. doi: 10.1377/hlthaff.2011.0639, 23048106

[ref32] Kajihara K. T. Hynson N. A. (2024). Networks as tools for defining emergent properties of microbiomes and their stability. Microbiome 12:184. doi: 10.1186/s40168-024-01868-z, 39342398 PMC11439251

[ref33] Kim S. Goel R. Kumar A. Qi Y. Lobaton G. Hosaka K. . (2018). Imbalance of gut microbiome and intestinal epithelial barrier dysfunction in patients with high blood pressure. Clin. Sci. (Lond.) 132, 701–718. doi: 10.1042/CS20180087, 29507058 PMC5955695

[ref34] Kim M. Huda M. N. Bennett B. J. (2022). Sequence meets function-microbiota and cardiovascular disease. Cardiovasc. Res. 118, 399–412. doi: 10.1093/cvr/cvab030, 33537709 PMC8803075

[ref35] Kurtz Z. D. Muller C. L. Miraldi E. R. Littman D. R. Blaser M. J. Bonneau R. A. (2015). Sparse and compositionally robust inference of microbial ecological networks. PLoS Comput. Biol. 11:e1004226. doi: 10.1371/journal.pcbi.1004226, 25950956 PMC4423992

[ref36] Lee D. M. Ecton K. E. Trikha S. R. J. Wrigley S. D. Thomas K. N. Battson M. L. . (2020). Microbial metabolite indole-3-propionic acid supplementation does not protect mice from the cardiometabolic consequences of a Western diet. Am. J. Physiol. Gastrointest. Liver Physiol. 319, G51–G62. doi: 10.1152/ajpgi.00375.2019, 32421360 PMC7468755

[ref37] Lee S. Y. Michell K. A. Butler M. M. Smith B. T. Woolf E. K. Holmes S. C. . (2025). Feasibility and tolerability of daily microgreen consumption in healthy middle-aged/older adults: a randomized, open-label, controlled crossover trial. Nutrients 17:467. doi: 10.3390/nu17030467, 39940327 PMC11820112

[ref38] Lewinsohn T. M. Inácio Prado P. Jordano P. Bascompte J. Olesen M. (2006). Structure in plant–animal interaction assemblages. Oikos 113, 174–184. doi: 10.1111/j.0030-1299.2006.14583.x

[ref39] Li J. Zhao F. Wang Y. Chen J. Tao J. Tian G. . (2017). Gut microbiota dysbiosis contributes to the development of hypertension. Microbiome 5:14. doi: 10.1186/s40168-016-0222-x, 28143587 PMC5286796

[ref40] Lin H. Peddada S. D. (2020). Analysis of compositions of microbiomes with bias correction. Nat. Commun. 11:3514. doi: 10.1038/s41467-020-17041-7, 32665548 PMC7360769

[ref41] Liu C. Cui Y. Li X. Yao M. (2020). Microeco: an R package for data mining in microbial community ecology. FEMS Microbiol. Ecol. 97:fiaa255. doi: 10.1093/femsec/fiaa255, 33332530

[ref42] Liu H. Feng S. Tang M. Tian R. Zhang S. (2024). Gut commensal *Bacteroides thetaiotaomicron* promote Atherothrombosis via regulating L-tryptophan metabolism. Rev. Cardiovasc. Med. 25:395. doi: 10.31083/j.rcm2511395, 39618850 PMC11607515

[ref43] Liu C. Li C. Jiang Y. Zeng R. J. Yao M. Li X. (2023). A guide for comparing microbial co-occurrence networks. iMeta 2:e71. doi: 10.1002/imt2.71, 38868345 PMC10989802

[ref44] Liu C. Mansoldo F. R. Li H. Vermelho A. B. Zeng R. J. Li X. . (2025). A workflow for statistical analysis and visualization of microbiome omics data using the R microeco package. Nat. Protoc. 21, 1300–1324. doi: 10.1038/s41596-025-01239-4, 40770112

[ref45] Liu L. Zhou Q. Xu T. Deng Q. Sun Y. Fu J. . (2025). Non-differential gut microbes contribute to hypertension and its severity through co-abundances: a multi-regional prospective cohort study. iMeta 4:e268. doi: 10.1002/imt2.268, 40027484 PMC11865328

[ref46] Lopez-Montoya P. Cerqueda-Garcia D. Rodriguez-Flores M. Lopez-Contreras B. Villamil-Ramirez H. Moran-Ramos S. . (2022). Association of gut microbiota with atherogenic dyslipidemia, and its impact on serum lipid levels after bariatric surgery. Nutrients 14:3545. doi: 10.3390/nu14173545, 36079803 PMC9460232

[ref47] Louca P. Nogal A. Wells P. M. Asnicar F. Wolf J. Steves C. J. . (2021). Gut microbiome diversity and composition is associated with hypertension in women. J. Hypertens. 39, 1810–1816. doi: 10.1097/HJH.0000000000002878, 33973959 PMC7611529

[ref48] Love M. I. Huber W. Anders S. (2014). Moderated estimation of fold change and dispersion for RNA-seq data with DESeq2. Genome Biol. 15:550. doi: 10.1186/s13059-014-0550-8, 25516281 PMC4302049

[ref49] Lu Y. Zhou G. Ewald J. Pang Z. Shiri T. Xia J. (2023). MicrobiomeAnalyst 2.0: comprehensive statistical, functional and integrative analysis of microbiome data. Nucleic Acids Res. 51, W310–W318. doi: 10.1093/nar/gkad407, 37166960 PMC10320150

[ref50] Martin B. D. Witten D. Willis A. D. (2020). Modeling microbial abundances and dysbiosis with beta-binomial regression. Ann. Appl. Stat. 14, 94–115. doi: 10.1214/19-aoas1283, 32983313 PMC7514055

[ref51] McCrea C. E. Skulas-Ray A. C. Chow M. West S. G. (2012). Test-retest reliability of pulse amplitude tonometry measures of vascular endothelial function: implications for clinical trial design. Vasc. Med. 17, 29–36. doi: 10.1177/1358863X11433188, 22363016 PMC3513268

[ref52] Morales C. Rojas G. Rebolledo C. Rojas-Herrera M. Arias-Carrasco R. Cuadros-Orellana S. . (2022). Characterization of microbial communities from gut microbiota of hypercholesterolemic and control subjects. Front. Cell. Infect. Microbiol. 12:943609. doi: 10.3389/fcimb.2022.943609, 36523636 PMC9745040

[ref53] Natarajan N. Hori D. Flavahan S. Steppan J. Flavahan N. A. Berkowitz D. E. . (2016). Microbial short chain fatty acid metabolites lower blood pressure via endothelial G protein-coupled receptor 41. Physiol. Genomics 48, 826–834. doi: 10.1152/physiolgenomics.00089.2016, 27664183 PMC6223570

[ref54] Nearing J. T. Douglas G. M. Hayes M. G. MacDonald J. Desai D. K. Allward N. . (2022). Microbiome differential abundance methods produce different results across 38 datasets. Nat. Commun. 13:342. doi: 10.1038/s41467-022-28034-z, 35039521 PMC8763921

[ref55] Nogal A. Valdes A. M. Menni C. (2021). The role of short-chain fatty acids in the interplay between gut microbiota and diet in cardio-metabolic health. Gut Microbes 13, 1–24. doi: 10.1080/19490976.2021.1897212, 33764858 PMC8007165

[ref56] Paulson J. N. Stine O. C. Bravo H. C. Pop M. (2013). Differential abundance analysis for microbial marker-gene surveys. Nat. Methods 10, 1200–1202. doi: 10.1038/nmeth.2658, 24076764 PMC4010126

[ref57] Peters B. A. Xue X. Hanna D. B. Wang Y. Wang Z. Sharma A. . (2025). Healthy aging and the gut microbiome in people with and without HIV. J. Infect. Dis. 231, 981–992. doi: 10.1093/infdis/jiae644, 39841165 PMC11998545

[ref58] Pinart M. Dotsch A. Schlicht K. Laudes M. Bouwman J. Forslund S. K. . (2021). Gut microbiome composition in obese and non-obese persons: a systematic review and meta-analysis. Nutrients 14:12. doi: 10.3390/nu14010012, 35010887 PMC8746372

[ref59] Pluznick J. (2014). A novel SCFA receptor, the microbiota, and blood pressure regulation. Gut Microbes 5, 202–207. doi: 10.4161/gmic.27492, 24429443 PMC4063845

[ref60] Pluznick J. L. Protzko R. J. Gevorgyan H. Peterlin Z. Sipos A. Han J. . (2013). Olfactory receptor responding to gut microbiota-derived signals plays a role in renin secretion and blood pressure regulation. Proc. Natl. Acad. Sci. USA 110, 4410–4415. doi: 10.1073/pnas.1215927110, 23401498 PMC3600440

[ref61] Powell-Wiley T. M. Poirier P. Burke L. E. Despres J. P. Gordon-Larsen P. Lavie C. J. . (2021). Obesity and cardiovascular disease: a scientific statement from the American Heart Association. Circulation 143, e984–e1010. doi: 10.1161/CIR.0000000000000973, 33882682 PMC8493650

[ref62] Roth G. A. Mensah G. A. Johnson C. O. Addolorato G. Ammirati E. Baddour L. M. . (2020). Global burden of cardiovascular diseases and risk factors, 1990-2019: update from the GBD 2019 study. J. Am. Coll. Cardiol. 76, 2982–3021. doi: 10.1016/j.jacc.2020.11.010, 33309175 PMC7755038

[ref63] Sears C. L. Pardoll D. M. (2011). Perspective: alpha-bugs, their microbial partners, and the link to colon cancer. J. Infect. Dis. 203, 306–311. doi: 10.1093/jinfdis/jiq061, 21208921 PMC3071114

[ref64] Sinha T. Vich Vila A. Garmaeva S. Jankipersadsing S. A. Imhann F. Collij V. . (2019). Analysis of 1135 gut metagenomes identifies sex-specific resistome profiles. Gut Microbes 10, 358–366. doi: 10.1080/19490976.2018.1528822, 30373468 PMC6546312

[ref65] Skye S. M. Zhu W. Romano K. A. Guo C. J. Wang Z. Jia X. . (2018). Microbial transplantation with human gut commensals containing CUTC is sufficient to transmit enhanced platelet reactivity and thrombosis potential. Circ. Res. 123, 1164–1176. doi: 10.1161/CIRCRESAHA.118.313142, 30359185 PMC6223262

[ref66] Sun S. Lulla A. Sioda M. Winglee K. Wu M. C. Jacobs D. R. . (2019). Gut microbiota composition and blood pressure. Hypertension 73, 998–1006. doi: 10.1161/HYPERTENSIONAHA.118.12109, 30905192 PMC6458072

[ref67] Sun H. J. Wu Z. Y. Nie X. W. Bian J. S. (2019). Role of endothelial dysfunction in cardiovascular diseases: the link between inflammation and hydrogen sulfide. Front. Pharmacol. 10:1568. doi: 10.3389/fphar.2019.01568, 32038245 PMC6985156

[ref68] Tang T. W. H. Chen H. C. Chen C. Y. Yen C. Y. T. Lin C. J. Prajnamitra R. P. . (2019). Loss of gut microbiota alters immune system composition and cripples Postinfarction cardiac repair. Circulation 139, 647–659. doi: 10.1161/CIRCULATIONAHA.118.035235, 30586712

[ref69] Tang W. H. W. Hazen S. L. (2024). Unraveling the complex relationship between gut microbiome and cardiovascular diseases. Circulation 149, 1543–1545. doi: 10.1161/CIRCULATIONAHA.123.067547, 38739698 PMC11095831

[ref70] Tjalsma H. Boleij A. Marchesi J. R. Dutilh B. E. (2012). A bacterial driver-passenger model for colorectal cancer: beyond the usual suspects. Nat. Rev. Microbiol. 10, 575–582. doi: 10.1038/nrmicro2819, 22728587

[ref71] Trikha S. R. J. Lee D. M. Ecton K. E. Wrigley S. D. Vazquez A. R. Litwin N. S. . (2021). Transplantation of an obesity-associated human gut microbiota to mice induces vascular dysfunction and glucose intolerance. Gut Microbes 13:1940791. doi: 10.1080/19490976.2021.1940791, 34313540 PMC8317959

[ref72] Tsao C. W. A. A. Almarzooq Z. I. Alonso A. Beaton A. Z. Bittencourt M. S. Boehme A. K. . (2022). Heart disease and stroke statistics — 2022 update: a report from the American Heart Association. Circulation. 10.1161/CIR.0000000000001052 35078371

[ref73] Vikram A. Kim Y. R. Kumar S. Li Q. Kassan M. Jacobs J. S. . (2016). Vascular microRNA-204 is remotely governed by the microbiome and impairs endothelium-dependent vasorelaxation by downregulating Sirtuin1. Nat. Commun. 7:12565. doi: 10.1038/ncomms12565, 27586459 PMC5025761

[ref74] Walker R. L. Vlamakis H. Lee J. W. J. Besse L. A. Xanthakis V. Vasan R. S. . (2021). Population study of the gut microbiome: associations with diet, lifestyle, and cardiometabolic disease. Genome Med. 13:188. doi: 10.1186/s13073-021-01007-5, 34915914 PMC8680346

[ref75] Wang Z. Usyk M. Vazquez-Baeza Y. Chen G. C. Isasi C. R. Williams-Nguyen J. S. . (2021). Microbial co-occurrence complicates associations of gut microbiome with US immigration, dietary intake and obesity. Genome Biol. 22:336. doi: 10.1186/s13059-021-02559-w, 34893089 PMC8665519

[ref76] Wang J. Wang C. Zhang J. Wu X. Hou Y. Zhao G. . (2022). Decreased precipitation reduced the complexity and stability of bacterial co-occurrence patterns in a semiarid grassland. Front. Microbiol. 13:1031496. doi: 10.3389/fmicb.2022.1031496, 36620016 PMC9815162

[ref77] WHO Cardiovascular diseases (CVDs) World Health Organization (2017) Available online at: https://www.who.int/news-room/fact-sheets/detail/cardiovascular-diseases-(cvds)

[ref78] Wilmanski T. Diener C. Rappaport N. Patwardhan S. Wiedrick J. Lapidus J. . (2021). Gut microbiome pattern reflects healthy ageing and predicts survival in humans. Nat. Metab. 3, 274–286. doi: 10.1038/s42255-021-00348-0, 33619379 PMC8169080

[ref79] Wu M. H. Chen S. Y. Chen J. W. Xue K. Chen S. L. Wang X. M. . (2021). Reduced microbial stability in the active layer is associated with carbon loss under alpine permafrost degradation. Proc. Natl. Acad. Sci. USA 118. doi: 10.1073/pnas.2025321118, 34131077 PMC8237688

[ref80] Wu C. Yang F. Zhong H. Hong J. Lin H. Zong M. . (2024). Obesity-enriched gut microbe degrades myo-inositol and promotes lipid absorption. Cell Host Microbe 32, 1301–1314.e9. doi: 10.1016/j.chom.2024.06.012, 38996548

[ref81] Xia M. Xu Y. Li H. Huang J. Zhou H. Gao C. . (2024). Structural and functional alteration of the gut microbiota in elderly patients with hyperlipidemia. Front. Cell. Infect. Microbiol. 14:1333145. doi: 10.3389/fcimb.2024.1333145, 38812752 PMC11133514

[ref82] Xiao M. Zhang C. Duan H. Narbad A. Zhao J. Chen W. . (2024). Cross-feeding of bifidobacteria promotes intestinal homeostasis: a lifelong perspective on the host health. NPJ Biofilms and Microbiomes 10:47. doi: 10.1038/s41522-024-00524-6, 38898089 PMC11186840

[ref83] Xu H. Fang F. Wu K. Song J. Li Y. Lu X. . (2023). Gut microbiota-bile acid crosstalk regulates murine lipid metabolism via the intestinal FXR-FGF19 axis in diet-induced humanized dyslipidemia. Microbiome 11:262. doi: 10.1186/s40168-023-01709-5, 38001551 PMC10675972

[ref84] Yang L. Chen J. (2022). A comprehensive evaluation of microbial differential abundance analysis methods: current status and potential solutions. Microbiome 10:130. doi: 10.1186/s40168-022-01320-0, 35986393 PMC9392415

[ref85] Zheng J. An Y. Du Y. Song Y. Zhao Q. Lu Y. (2024). Effects of short-chain fatty acids on blood glucose and lipid levels in mouse models of diabetes mellitus: a systematic review and network meta-analysis. Pharmacol. Res. 199:107041. doi: 10.1016/j.phrs.2023.107041, 38128856

