# Supplementary material for: Gut microbiome dynamics and cardiometabolic disease progression: evidence from a secondary data analysis of pooled clinical datasets
Source: Front Microbiol. 2026 Jul 27;17:1790805. doi: 10.3389/fmicb.2026.1790805 (PMC13455506; doi:10.3389/fmicb.2026.1790805)
Supplement: Supplementary file 1 [file Supplementary_file_1.DOCX]

Supplementary Material

Supplementary Figure 1. Box plots of phyla level Shannon’s and Chao1 diversity indices for clinical stratifications of BP (A, B), RHI (C, D), LDL (E, F), TC (G, H), and BMI (I, J) revealed no statistically significant differences in alpha diversity. Shannon’s diversity indices were analyzed utilizing nonparametric Mann-Whitney/Kruskal-Wallis statistical tests. Statistical significance was assigned as p<0.05 and a false discovery rate <0.05. *BP = blood pressure; RHI = reactive hyperemia index; LDL = low density lipoprotein; TC = total cholesterol; BMI = body mass index.*

Supplementary Figure 2. PCoA of phyla level Bray-Curtis distances showed no significant separation of the gut microbiota across clinical stratifications of BP (A), RHI (B), LDL (C), TC (D), or BMI (E), as calculated by PERMANOVA. Statistical significance was assigned as p<0.05 and a false discovery rate <0.05. *BP = blood pressure; RHI = reactive hyperemia index; LDL = low-density lipoprotein; TC = total cholesterol; BMI = body mass index.*

**Supplementary Figure 3.** Heatmaps showing correlations of differentially abundant taxa identified in at least two comparisons with systolic BP (**A**), diastolic BP (**B**), RHI (**C**), LDL (**D**), TC (**E**), and BMI (**F**). Statistical significance was assigned as p<0.05 and an adjusted p-value of <0.1. *BP = blood pressure; RHI = reactive hyperemia index; LDL = low-density lipoprotein; TC = total cholesterol; BMI = body mass index.*

**Supplementary Figure 4.** The total of absolute links at the phylum level. *BP = blood pressure; RHI = reactive hyperemia index; LDL = low-density lipoprotein; TC = total cholesterol; BMI = body mass index.*

**Supplementary Figure 5.** The relative proportion of linked nodes connected through positive and negative edges across networks at the phylum level for BP (**A**), RHI (**B**), LDL (**C**), TC (**D**), and BMI (**E**). Phyla with a mean relative contribution <0.01 to positive or negative edges were removed for visualization. *BP = blood pressure; LDL = low-density lipoprotein; TC = total cholesterol; RHI = reactive hyperemia index; BMI = body mass index.*
